# Long-term hydrological drought monitoring and trend analysis in Blue Nile River basin

**DOI:** 10.1016/j.heliyon.2024.e41161

**Published:** 2024-12-12

**Authors:** Kassa Abera Tareke

**Affiliations:** Department of Hydraulics and Water Resource Engineering, Kombolcha Institute of Technology, KioT, Wollo University, Ethiopia

**Keywords:** Hydrological drought, Mann-kendall test, Streamflow drought index (SDI), Trend analysis, Riparian countries

## Abstract

This research aims to monitor the hydrological drought trends within the geographical confines of Ethiopia, Sudan, and Egypt in the Blue Nile River Basin. Historical drought circumstances in the basin were analyzed through the utilization of the stream flow drought index (SDI). The long-term historical drought trend was investigated via the application of the Mann – Kendall Sen (MK) test. Streamflow data were collected at the border (GERD) (Ethiopia) and Khartoum and Dongola (Sudan) spanning the period from 1900 to 2001. Four distinct temporal scales were examined, including monthly (SDI1), seasonal (SDI3), bi-annual (SDI6), and annual (SDI12) frequency. Notably, SDI1, SDI3, and SDI6 exhibited a higher frequency of drought occurrences, whereas SDI12 demonstrated lower frequencies, accompanied by the longest duration of drought in all gauged stations. For the preceding 102-year period, two extreme drought events were identified across all stations: 1912/1913 and 1913/1914 in the Border and Dongola stations, and 1912/1913 and 1986/1987 in the Khartoum station. Moreover, the SDI12 results revealed that severe drought events manifested three, six, and four times, in the Border, Khartoum, and Dongola stations, respectively. Furthermore, an investigation of historical extreme and severe drought patterns led to the conclusion that extreme hydrological drought does not pose an imminent threat to downstream nations, including Egypt and Sudan. However, the trend analysis revealed that an increasing drought trend was observed in the Autumn season across all stations, while a positive trend characteristic of a wet condition was observed in the remaining seasons. Annual trend analysis did not show any statistically significant findings. Nevertheless, the study highlighted the imperative role of soil and water conservation measures in upstream countries, such as the Ethiopian highlands, in mitigating the prolonged effects of meteorological drought which gradually propagates into severe hydrological drought. Consequently, downstream nations must engage in cooperative efforts with upstream countries to address this issue collectively, rather than bestowing sole responsibility on the latter.

## 1 Introduction

Drought is a natural hazard, globally it has a high impact on social, environmental, and economic [1]. Hydrological extreme events both high flow (flood) and low flow (drought) are recently the basic issues in the world [2]. Contrarily, compared to other catastrophic events, drought is one of the most frequent disasters that have a significant detrimental effect on water resource activities including irrigation, hydropower, and water supply [3].

Almost all topographic and climatic zones are susceptible to drought, and the severity, duration, and other aspects of the condition vary greatly from one place to another [4]. It is difficult to adapt the definition of drought as well as the impact associated with drought due to the interrelationship between different hydroclimatic variables such as ecology, geology, hydrologic, climate, and socioeconomic factors in a specific study area [4,5]. The effect of drought can be continued for some period, it may take a year or more after termination due to its gradual propagation from moderate to severe stage [4]. Because of this, drought has no common universal definition [5,6]. However, droughts can be (1) meteorological, which is the scarcity of precipitation, (2) hydrological drought which is associated with the scarcity of streamflow, decrease in surface and subsurface water (Lake and reservoir volume) and groundwater level and, (3) agricultural drought related to a reduction in available of soil moisture for crop production and (4) socio-economical drought refers to high demand on natural goods due to less supply and each drought is a cause of the other [6,7].

Most drought studies in East Africa were focused on meteorological drought and somehow agricultural drought [[8], [9], [10], [11], [12], [13], [14], [15]]. Few research studies are conducted in Northern and Eastern and some parts of the Blue Nile in Ethiopia which are mainly focused on meteorological drought analysis [[8], [9], [10]].

Hydrological drought has received minimal attention in previous research investigations, which have focused more on meteorological drought [16]. However, hydrological drought has drawn more attention in recent years due to its multidimensional impacts on the socio-economic and overall ecosystem [17]. For example, recent studies found that hydrological droughts had a greater overall impact than other forms of droughts [18,19]. According to Ref. [17], hydrological droughts have a significant impact on agriculture, energy, navigation, water supply and recreation, shipping, and fisher sectors compared to other types of droughts such as meteorological droughts. From this, it is understood that further investigation of hydrological drought monitoring and impact assessment is important to develop a comprehensive drought mitigation measure. Therefore, the objective of this study is (1) to analyze long-term hydrological drought severity using SDI, (2) trend analysis using the Mann – Kendall test, (3) to develop good recommendations based on the severity and trend result for stakeholders, water resource managers, and policymakers as well as water riparian countries for equitable utilization of surface water. Non-transboundary river basins such as Awash Basin have relatively studied both the long-term variability of rainfall and streamflow and the associated impact on agriculture and food security issues [20,21]. Blue Nile is an umbrella for many people for their food security and it is not easy to feed themselves using rainfed agriculture. To upgrade the production capacity of rainfed agriculture, proper utilization of surface and groundwater resources using irrigation is vital in the basin which is recurrently affected by drought [22,23].

It is widely acknowledged that to effectively address the risk of hydrological drought, a change from a reactive to a proactive approach is required. However, such a shift in drought management necessitates the backing of several strategies and tactics that can be used for both planning and carrying out mitigation actions [24]. In developed countries, the problem related to water is an unbalance between water resources and demand due to population increase, urbanization, tourism growth, and irrigation and agriculture enlargement. But in developing countries, even though there are water resource constraints in many countries, the main problem is hydrological events such as floods and droughts. Drought is the most critical issue in East Africa and needs a good investigation in early warning development and drought mitigation measurement [[25], [26], [27], [28]].

In East Africa, such as Egypt and Sudan the precipitation is very erratic, and it is difficult to analyze the meteorological drought impact [[29], [30], [31]]. The regions are already dry areas and receive low rainfall and the main source of water is the Nile River which receives water from the Blue Nile and White Nile. Blue Nile originated from Ethiopia and it contributes 86 % [32] of surface water to the downstream countries. [32], reviewed ten different research papers on East Blue Nile related to drought, all are meteorological drought monitoring using SPI and other indices. Therefore, this study is focused on long-term hydrological drought monitoring and trend analysis in the Blue Nile River basin using the streamflow drought index (SDI), and the trend was analyzed using the Mann – Kendall test. Recently, most riparian counties in the Blue Nile have an increased interest in conserving surface water for different agricultural and industrial scale-up purposes to improve their society's livelihood standards by constructing dams and creating large reservoirs. However, irrigation, energy, tourism, and other surface and groundwater resource-dependent sectors are highly susceptible to hydrological drought impact. Therefore, countries sharing a transboundary river have to develop common drought management policies and integrated water resource management practices to reduce the hydrological drought impact on human life and the overall development of the countries.

In this particular study, the methodology may be similar to other previous studies but this study covers a large area, and hydrological drought analysis is not well investigated in the region, the Blue Nile River is transboundary more than 10 countries share the resource and it is sensitive for conflict during drought events. Especially Ethiopia, Sudan, and Egypt are the most vulnerable countries for the issue of water sharing during drought and it is important to show the trend of hydrological drought in the region to shift the attitude of leaders from dispute agenda to how to manage, equitable utilize, conservation and development of this natural resource in a cooperative framework. So, this study's findings have good insights for researchers, politicians, and journalists with a scientific justification of the historical and future trend of hydrological drought in the region and it opens a door for future researchers on how to shift cooperatively from reactive to proactive paradigm of drought mitigation and adaptation mechanism within the riparian countries.

## 2 Methods and materials

### 2.1 Study area description

**Fig. 1:**
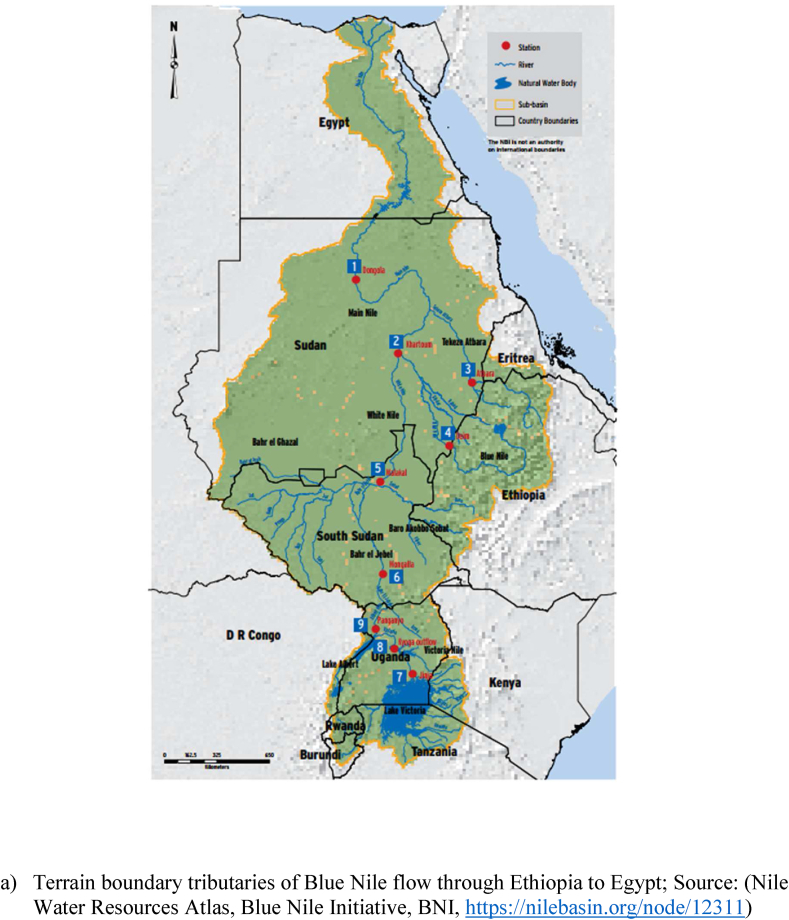

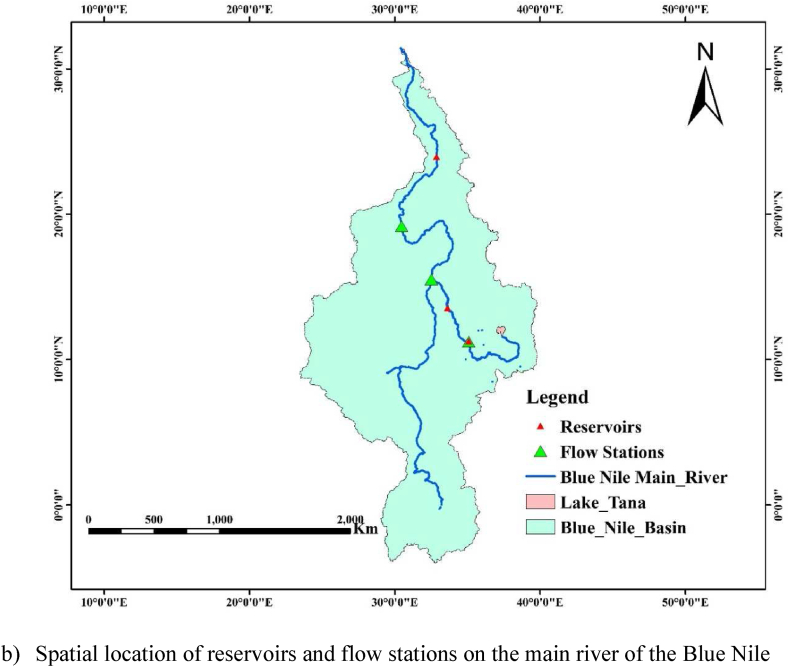
Terrian map (a) and spatial location of hydrometeorological station (b) in the Blue Nile River basin; from Ethiopia to Egypt.

Blue Nile River is the longest river in the world with an approximate length of 1450 km. Blue Nile River originated from Lake Tana, Ethiopia and the main tributaries originated from Ethiopian highlands. The main tributaries for the Blue Nile are the Tekeze River, Baro Akobo River, and Abbay Rivers, which account for 86 % of the Nile basin river flow and the remaining 14 % is from the White Nile. As indicated in Figure (1a and b), the two rivers (Blue and White Nile) joined at Khartoum, Sudan.

This study covers a large area of Ethiopian highlands with a maximum rainfall of above 2000 mm and a minimum rainfall of 300 mm, Sudan has a maximum of 1200 mm and a minimum of 100 mm, and Egypt has a maximum of 200 mm and a minimum of 20 mm respectively. The mean annual temperature ranges from 15 to 25 ^o^c, 23–32 ^o^c. and 15–32 ^o^c in Ethiopia, Sudan, and Egypt respectively. The most known reservoirs that exist in the main river line of the Blue Nile are GERD (Ethiopia), Sennar (Sudan), and Aswan (Egypt) as shown in Fig. 1. These reservoirs have a significant role in regulating the flow and the sediment transportation from upstream to downstream countries. Sudan and Egypt are located in the low land areas compared to Ethiopia and their probability of affecting by hydrological events like floods is maximum. Therefore, the presence of these cascade reservoirs can reduce the possibility of flood occurrence and limit its expansion out of the main river bank. However, it is important to investigate the impact of drought on reservoir water allocation while severe and extreme drought occurs. For instance, this kind of drought monitoring and trend analysis has a vital role in integrated water resource management for riparian countries. Blue Nile River in Ethiopia has 16 sub-basins with more than 20 tributary rivers. However, this study is focused on the main river flow condition from Ethiopia to Egypt (upstream to downstream).

### 2.2 Hydrological data

The long-term hydrological data (streamflow) was collected from the Ministry of Water and Energy (MoWE), Ethiopia in three stations (1900–2001) as shown in Table 1. Streamflow data of the three stations is interpolated from different nearby stations for Grand Ethiopian Renaissance Dam (GERD) analysis by the three countries (Ethiopia, Sudan, and Egypt). Then all those Countries may have this data. Even though the data is interpolated from the nearby stations, now it is applied for design purposes for GERD and different reservoirs in Sudan. Therefore, this interpolated data can also be used here for hydrological drought analysis in the Blue Nile River to investigate the historical drought severity and trend in this river basin. Of course, this interpolated data will have some accuracy problems the historical trend indicates the low flow and maximum flow occurrence in the same seasons and years as supported by different researchers in the Blue Nile River basin [[33], [34], [35], [36]]. The minimum annual mean flow occurred during 1913 for all stations but the maximum annual flow occurred during 1909 for both border at GERD and Khartoum stations and 1998 for Dongola (Table 1 a and b).

**Table 1:** Monthly (a) and long-term mean annual streamflow (b) of selected stations in Ethiopia and Sudan (1900–2001).

a) Monthly mean, maximum, and minimum flow data									
Month	Mean monthly flow (m3/s)								
	Border flow (m^3^/s)			Khartoum flow (m^3^/s)			Dongola flow (m^3^/s)		
	mean	max	min	mean	max	min	mean	max	min
Jan	306.3	731.3	69.9	307.7	728.0	108.3	1497.0	2453.0	642.2
Feb	201.0	566.0	58.2	194.4	558.0	69.4	1225.5	2497.9	475.4
Mar	144.4	404.7	51.5	133.5	392.0	33.6	991.3	2170.3	399.5
Apr	134.4	490.4	41.3	119.3	478.0	22.4	997.1	2028.9	366.5
May	219.5	691.6	68.8	197.0	682.9	56.0	909.0	1760.4	298.7
Jun	624.5	1570.0	150.5	605.0	1639.3	179.8	880.7	1892.4	347.2
Jul	2402.1	4704.3	853.5	2557.8	5199.0	701.9	1838.0	4117.8	649.6
Aug	5612.3	9270.6	2435.4	6170.5	9980.2	2807.6	6767.4	11073.4	2426.8
Sep	4724.0	8021.0	2315.1	5361.3	8834.9	2816.4	7887.2	11654.7	2820.6
Oct	2512.9	5187.1	787.3	2839.3	6011.1	991.3	5005.2	8512.5	2227.8
Nov	1044.8	1931.0	282.4	1103.7	2334.1	373.8	2901.9	5131.2	1567.1
Dec	536.3	1169.8	117.3	553.8	1179.8	203.9	1893.8	2942.1	1056.6

b) Annual mean, maximum, and minimum flow data							
No	Stations Name	Country	Latitude	Longitude	Mean flow (m^3^/s)	Maximum flow	Minimum flow
1	GERD	Ethiopia	11.22	35.09	1538.54	2293.34	643.16
2	Khartoum	Sudan	15.51	32.52	1678.61	2493.59	811.80
3	Dongola	Sudan	19.17	30.47	2732.85	3793.06	1446.27

**Fig. 2:**
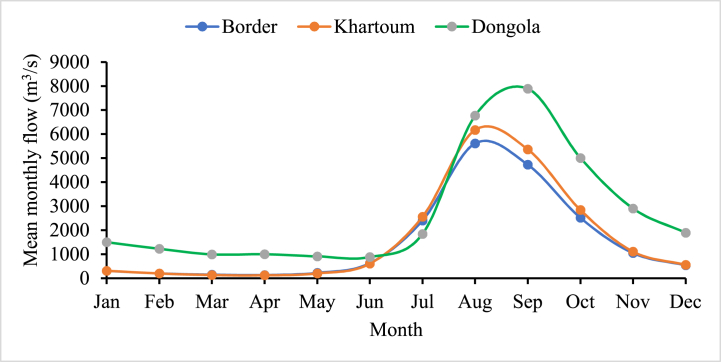
Mean monthly streamflow rate (m^3^/s) of selected stations from 1900 to 2001.

**Fig. 3:**
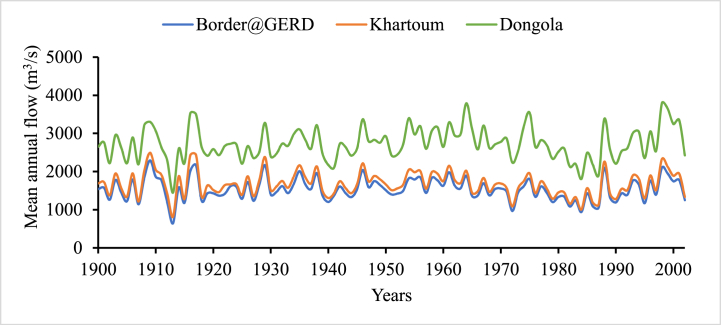
Mean annual streamflow rate (m3/s) time series of selected stations (1900–2001).

Fig. 2, Fig. 3 respectively, show the monthly and annual flow for Dongola station is maximum compared to GERD and Khartoum. Because it receives both the Blue Nile and White Nile streamflow below Khartoum. As a result, the commutative annual streamflow is increased due to the contribution of many tributaries.

### 2.3 Hydrological drought monitoring

In drought monitoring, several meteorological drought indices use precipitation and temperature as input data [[37], [38], [39], [40]]. However, in the case of agricultural and hydrological drought analysis, there are only a few indices. Some of the common hydrological droughts practiced in many regions are streamflow drought index (SDI) and surface water supply index (SWSI) [28,41]. The streamflow drought index (SDI) is the most robust and simple hydrological drought indicator which uses streamflow as input for analysis. In this study, SDI was applied to analyze the historical hydrological drought condition of the Blue Nile River basin using three streamflow stations data for 102 years (1900–2001) record. Meteorological drought indices do not take into consideration the water balance impact due to human interference in the natural hydrology phenomena such as irrigation hydropower and water supply. Whereas, hydrological drought considers such interactions in the natural phenomena and human impact on the natural flow [42].

#### 2.3.1 Streamflow drought index

Hydrological drought is not an easy task like meteorological drought analysis. It requires many input data in the hydrologic cycle. However, Nalbantis and Tsakiris (2009) [43] developed Streamflow Drought Index (SDI) which is a simple hydrological drought indicator using a single hydrological variable, streamflow [44,45]. Since its calculation is the same as SPI's, it shares the same traits of simplicity and effectiveness. To control hydrological drought and the supply of water in the short, monthly and seasonal (SDI1 and SDI3), medium, half-year (SDI6 and SDI9), and long-term, annual (SDI12 and above) time scales, the SDI is calculated based on monthly observed streamflow volumes at different time scales. The calculation is given by equation (1):

(1) $$Vi,j=\sum_{j=1}^{3k}Qi,j\;i=1,2\;\ldots \ldots ,j=1,2,\ldots 12\;\text{and}\;k=1,2,3,4$$

Where: V_i,j_ is the cumulative streamflow volume for the i-th hydrological year and the k-th reference period, K is the seasonal value (four seasons, Ethiopia case)

Q_i,j_ is monthly streamflow volume at the ith hydrological year and jth month within that year.

For each reference period k of the ith hydrological year, the Streamflow Drought Index (SDI) is defined as follows using the cumulative streamflow volumes Vi,k in equation (2):

(2) $$SDI=\frac{Vi,k-Vkm}{Sk}$$

Where: i = 1, 2; …, and k = 1, 2, 3, 4.

Where Vkm and sk are respectively the means and the standard deviation of cumulative streamflow volumes of the reference period k as these are estimated over a long period.

The range of wetness and dryness of SDI ranges between −2 and +2 [46,47]. The extremely dry and wet values are below −2 and above +2 respectively. The classification of the drought magnitude range of this index is described in Table 2.

**Table 2:** Hydrological drought category based on SDI values [43].

SDI value	Drought Categories
>2	Extreme wet
1.5–1.99	Severe wet
1.0–1.49	Moderate wet
−0.99–0.99	Near Normal
−1.0–−1.49	Moderate drought
−1.5–−1.99	Severe drought
< −2	Extreme drought

The main sources of tributary stream flow and rainfall for the Blue Nile River basin are from the upstream parts of the basin, Ethiopia. Therefore, the monthly, seasonal, biannual, and annual analysis of drought conditions depends on the situation of the Ethiopia season. So, the monthly, seasonal, and biannual SDI analyses are focused on August which is the month in which Ethiopia receives more rainfall and if there is low precipitation in this month, simultaneously the downstream countries Sudan and Egypt will receive low flow.

### 2.4 Drought trend analysis

#### 2.4.1 Mann – Kendall test-based drought trend analysis

Numerous methods are applied to detect drought trends in terms of streamflow and rainfall such as statistical approaches and rank-based tests. Statistical methods include Slope based tests, Least Squares Linear Regression (LR), Sen's Slope Estimator (SS), and Rank-based tests based on the Mann-Kendall (MK) test, Spearman Rank Correlation (SRC) test [48]. Pre-whitening, Trend pre-whitening, and Variance Correction with the Mann-Kendall test are considered in the serial correlation effect by statistical approaches [49]. In this study, the Mann-Kendall test is used to detect hydrological and meteorological drought trends at a 5 % significance level based on the value of SDI and SPI time sequences in all river basins by using the AUTO_MK_Sen.exe software.

The Mann-Kendall (MK) test is determined using two sequential time series (Xj and Xi) from n data points in a series. Each subsequent data value in a given time series is compared with other data of the series. The value of statistics S can be increased or decreased by 1 based on the rearrangement of the data sequence of earlier and later data values. Then the statistics S value is calculated using the two incremental and decrement data values net result. Therefore, the Mann-Kendall (MK) test statics S is estimated using Equations (3), (4) as stated below.

(3) $$S=\sum_{i=1}^{n-1}\sum_{j=i+1}^{n}Sign\left(Xj-Xi\right)$$

(4) $$Sign\left(Xj-Xi\right)=\left\{\begin{array}{c} 1\;;if\;Xj-Xi>0\\ 0;if\;Xj-Xi=0\\ -1;if\;Xj-Xi<0 \end{array}\right\}$$

Where: xj and xi are the values of data in years j and i respectively.

If there are fewer than ten data points in the time series, the value of |S| is compared directly to the distribution S. If there are more than ten data points, the value of the statistic S is distributed according to the mean and variance as shown in Equations (5), (6).

(5) E(S)=0

(6) $$Var\left(S\right)=\frac{m\left(-1\right)\left(2m+5\right)-\sum_{k=1}^{n}k1\left(k1-1\right)\left(2k1+5\right)}{18}$$

Where m and k_i_ are the number of SPI and SDI time series and the ties of the sample time series, respectively.

The performance of the Mann-Kendall test was computed by AUTO_MK_Sen.exe and the calculated statistics Z_c_ test is given by Equation (7).

(7) $$Zc=\left\{\begin{array}{c} \frac{S-1}{\sigma }\;;\;if\;S>0\\ 0;\;if\;S=0\\ \frac{S+1}{\sigma }\;;\;if\;S<0 \end{array}\right\}$$

**Fig. 4:**
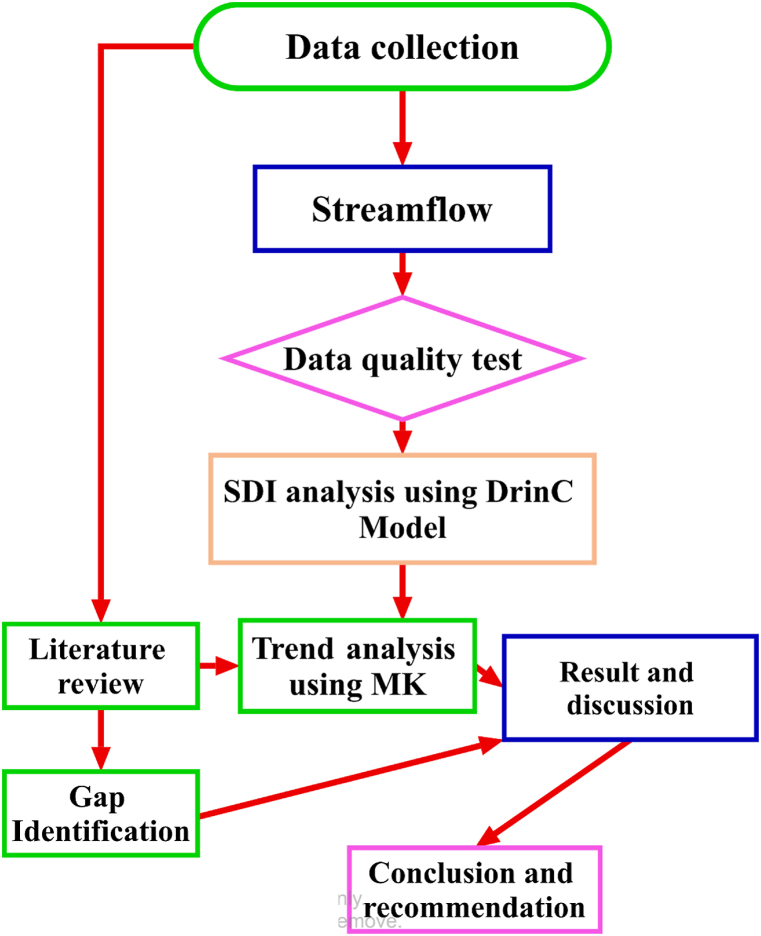
Overall activities and procedures diagram chart of the study.

The calculated Zc statistics follow a normal distribution and negative and positive Z_c_ values indicate a decreasing and increasing trend for a given time series period, respectively. The linear rate change (slope) and intercept of the normal distribution function in Z_c_ statistics are computed by Sen's Slope estimator test. In general, the overall activities and procedures of this study are summarized in Fig. 4.

## 3 Result and discussion

### 3.1 Result

#### 3.1.1 Long-term hydrological drought in the Blue Nile river basin

This study investigated the hydrological drought conditions for the previous 102 years in the Blue Nile River basin using the streamflow drought index (SDI). The analysis was checked with four time scales; monthly (SDI1), Seasonal (SDI3), biannual (SDI6), and annual (SDI12). The result indicates that the basin was frequently affected by moderate and severe drought for all time scales and the frequency of extreme drought was minimal for all stations. As shown in Table 3, the annual analysis (SDI12) value indicates that extreme drought occurred for stations at Border (GERD) and Dongola (Sudan) during 1912/13 and 1913/14 for two years. But for the case of Khartoum station, the extreme drought occurred during 1912/13 and 1986/87. From Table 3, the maximum duration of extreme drought for each station is 11, 9, and 7 months for Dongola, Border at GERD and Khartoum respectively.

**Table 3:** Identified moderate, severe, and extreme drought years for each station from 1900 to 2001.

Dongola Station Moderate, Severe and Extreme drought years and SDI12 values								
Moderate drought			Severe drought			Extreme drought		
Years	SDI	Duration (month)	Years	SDI	Duration (month)	Years	SDI	Duration (month)
1904/05	−1.31	5	1930/31	−1.60	9	1912/13	−2.49	9
1918/19	−1.18	4	1940/41	−1.81	6	1913/14	−2.85	11
1944/45	−1.10	4	1982/83	−1.75	5			
1972/73	−1.13	4	1986/87	−1.90	6			
1983/84	−1.23	3						
Total	5			4			2	

Border Station Moderate, Severe, and Extreme drought years and SDI values								
Moderate drought			Severe drought			Extreme drought		
Years	SDI	Duration(month)	Years	SDI	Duration (month)	Years	SDI	Duration (month)

1901/02	−1.11	3	1982/83	−1.69	7	1912/13	−3.45	7
1904/05	−1.21	3	1984/85	−1.71	7	1913/14	−2.16	9
1918/19	−1.05	5	1986/87	−1.98	7			
1940/41	−1.34	7						
1944/45	−1.20	3						
1971/72	−1.30	1	Percentage of drought occurrence (%)					
1972/73	−1.47	8	Station Name	Moderate		Severe	Extreme	
1980/81	−1.22	4	Border	12.7		2.9	1.9	
1981/82	−1.38	6	Khartoum	9.8		5.8	1.9	
1983/84	−1.43	6	Dongola	4.9		3.9	1.9	
1985/86	−1.03	5						
1989/90	−1.08	3						
1991/1992	−1.09	1						
Total	13			3			2	

Khartoum Station Moderate, Severe, and Extreme drought years and SDI values								
Moderate drought			Severe drought			Extreme drought		
Years	SDI	Duration(month)	Years	SDI	Duration (month)	Years	SDI	Duration (month)

1901/02	−1.11	2	1913/14	−1.70	10	1912/13	−2.84	4
1904/05	−1.30	3	1940/41	−1.50	8	1986/87	−2.03	7
1907/08	−1.02	2	1972/73	−1.53	8			
1944/45	−1.24	4	1982/83	−1.80	7			
1971/72	−1.21	1	1983/84	−1.56	6			
1980/81	−1.25	5	1984/85	−1.76	7			
1981/82	−1.44	3						
1985/86	−1.04	6						
1989/90	−1.16	2						
1991/92	−1.16	1						
Total	10			6			2	

**Fig. 5:**
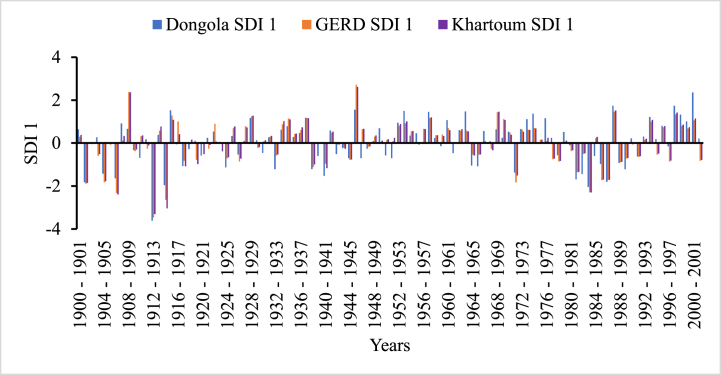
Monthly hydrological drought (SDI1) time series for GERD, Khartoum and Dongola stations, Blue Nile River Basin.

**Fig. 6:**
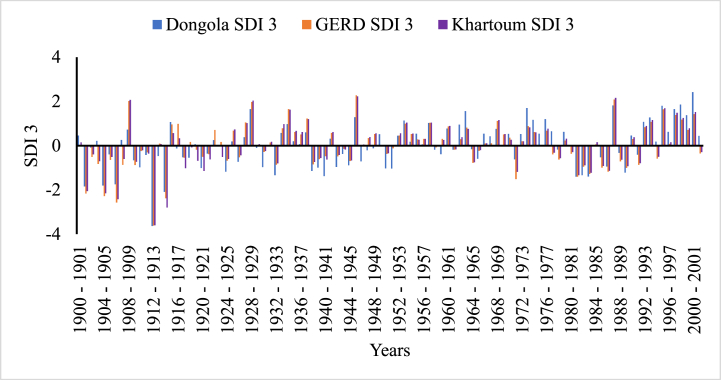
Seasonal hydrological drought (SDI3) time series for GERD, Khartoum and Dongola stations, Blue Nile River Basin.

**Fig. 7:**
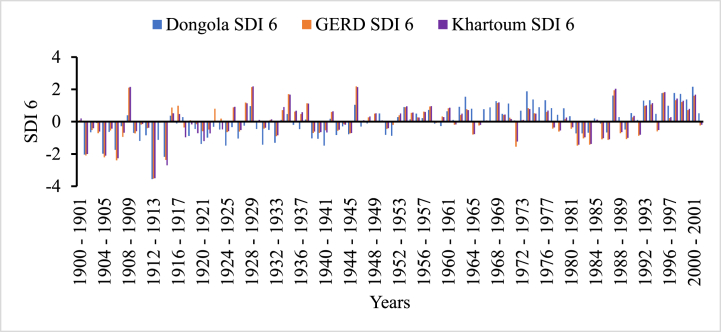
Bi-annual hydrological drought (SDI6) time series for GERD, Khartoum and Dongola stations, Blue Nile River Basin.

**Fig. 8:**
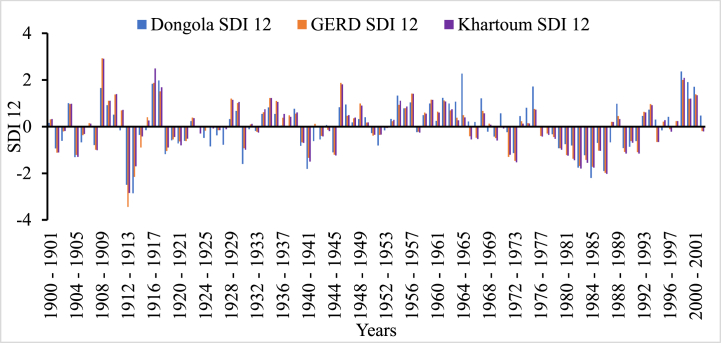
Annual hydrological drought (SDI12) time series for GERD, Khartoum and Dongola stations, Blue Nile River Basin.

In Fig. 5, Fig. 6, Fig. 7, Fig. 8, the frequency and magnitude of drought were high for SDI1, SDI3, and SDI6, and for the case of annual analysis (SDI12) both the frequency and magnitude decreased. For example, from 1901 to 1907, 1912–1914, and 1981–1987 the drought condition in the Blue Nile basin is found severe to extreme but for SDI12, the drought condition in 1981–1987 is moderate to severe for Border and Dongola stations extreme for Khartoum station. The frequency of moderate drought is higher in the upper parts of the Blue Nile basin (Ethiopia), but the severe and extreme drought conditions are minimal. The hydrological drought result here shows the frequency and magnitude of severe drought in the downstream countries is minimal compared to upper country Ethiopia. Because hydrological drought is dependent on the availability of surface and sub-surface water the downstream countries have a high chance to receive more river flow from different tributaries but, Ethiopia is the origin of many tributaries such as Tekeze, Baro, and Abbay. So, this study indicates that the downstream countries such as Egypt and Sudan have no significant threat related to hydrological drought.

The correlation between drought conditions of all time scales (SDI1, SDI3, SDI6, and SDI12) in the three selected stations is high (Table 4). Especially, border (GERD) and Khartoum station have a high correlation with R^2^ ranging from 0.98 to 0.99. This result implies the possibility of using SDI for hydrological drought monitoring in a large area and different hydroclimatic conditions.

**Table 4:** Correlation analysis of monthly, seasonal, half-year, and annual SDI values of selected streamflow stations (GERD, Khartoum and Dongola).

Correlation	Dongola SDI 1	GERD SDI1	Khartoum SDI 1
Dongola SDI 1	1	0.84	0.84
GERD SDI 1	0.84	1	**0.99**
Khartoum SDI 1	0.84	**0.99**	1

Correlation	Dongola SDI 3	GERD SDI 3	Khartoum SDI 3

Dongola SDI 3	1	0.86	0.87
GERD SDI 3	0.86	1	**0.98**
Khartoum SDI 3	0.87	**0.98**	1

Correlation	Dongola SDI 6	GERD SDI 6	Khartoum SDI 6

Dongola SDI 6	1	0.74	0.76
GERD SDI 6	0.74	1	**0.98**
Khartoum SDI 6	0.76	**0.98**	1

Correlation	Dongola SDI 12	GERD SDI 12	Khartoum SDI 12

Dongola SDI 12	1	0.87	0.87
GERD SDI 12	0.87	1	**0.99**
Khartoum SDI 12	0.87	**0.99**	1

The climate conditions and topography of the upstream country (Ethiopia) differ from those of downstream countries such as Sudan and Egypt. However, the input of SDI is streamflow which flows from upstream to downstream for a long distance within some reduction or increase depending on the number of tributaries and river area. Meteorological drought monitoring indices are limited to a specific region due to rainfall and temperature variability. The erratic rainfall in Egypt and Sudan will affect the results of any analysis of the Blue Nile River's meteorological drought condition using precipitation data from Ethiopia, Sudan, and Egypt. The main factor in hydrological drought analysis using SDI is human intervention on the river by diverting the streamflow, storage using a reservoir, and other purposes that hurt the streamflow condition upstream and downstream of the intervention point.

#### 3.1.2 Hydrological drought trend

Drought results from a deficiency of precipitation for a prolonged period in a specific area. Its trend is directly associated with the trend of precipitation and streamflow variation. The monthly, seasonal, and annual trend analysis of hydrological drought for three streamflow stations were computed using the Mann-Kendall (MK) test and the Sen slope estimator integrated with AUT_MK_Sen.exe software which is freely downloaded from the website:https://drive.google.com/file/d/1HVP81cxEpkPwf9qksCiV7W9p9bwXJ/view.

The result indicated that there is no significant annual trend in all stations but there is a significant increasing and decreasing trend in monthly and seasonal time scales (see Table 5). In the Blue Nile basin, the three streamflow stations show that there is an increasing drought trend for the months, of September, October, November, and December with a Z_calculated_ value of −2.22 to −3.71 (see Table 5). In a seasonal SDI analysis, only Autumn (September to November) shows an increasing drought trend and the remaining seasons have a positive significant trend, which is a high wet trend. Autumn is a season next to Summer (Kiremt), the distribution of rainfall in most parts of the basin in the upstream country, Ethiopia is very low and erratic. Consequently, the streamflow discharge radically decreased which has a direct impact on reservoirs and the production of irrigation and hydropower projects. Therefore, integrated water resource management practice has to be implemented in the upstream countries to increase the potential of springs and streams that join the main Blue Nile River. Egypt and Sudan are the most beneficiary downstream countries of the Blue Nile River. However, they are always complaining about projects implemented by Ethiopia rather than cooperatively involved in the management practices of the basin. So, riparian countries have to cooperate to develop good water resource management policies for equitable utilization in a sustainable manner.

**Table 5:** Mann – Kendall (MK) test for seasonal and annual SDI values from 1900 to 2001.

Stations	Seasonal Hydrological drought trend (Z- Value)								
	Sep	Oct	Nov	Dec	Jan	Feb	Mar	Apr	May
GERD	**−3.21**	−1.5	−1.92	−1.82	−1.46	−0.97	**−2.16**	−0.78	0.17
Khartoum	**−3.71**	**−2.36**	**−2.22**	**−2.26**	−2.1	−1.37	**−3.08**	−1.73	0.97
Dongola	**−2.48**	**−3.05**	**−2.79**	−0.85	1.8	**2.91**	**3.29**	**2.59**	**2.66**

Stations	Jun	Jul	Aug	Autumn	Winter	Spring	Summer	Annual
GERD	1.84	**2.38**	0.68	**−2.84**	−1.54	−1.55	1.83	−0.86
Khartoum	**3.07**	**3.01**	0.7	**−2.71**	**−1.98**	−1.87	**2.39**	−1.03
Dongola	**3.65**	**3.42**	1.39	**−2.45**	**1.98**	**2.64**	**2.96**	0.58

Note: numbers written in bold indicate the positive and negative significance (wet and dry trends), respectively.

Fig. 8 indicates the hydrological drought trend of Border (at GERD), Khartoum, and Dongola stations for monthly, seasonal, and annual time scales. The classification of the season is the same as the upstream country, Ethiopia, Autumn from September–November, Winter from December–February, Spring from March–May, and Summer from June–August. It is observed that the drought has a significant trend from September to March and a wet trend from April to August. In the case of seasonal time scale, Autumn has a dry trend and the remaining three seasons (winter, spring, and summer) have a wet trend. The annual time scale analysis does not show any trend. Since Dongola received flow from White Nile as a result its drought trend is somehow positive relative to GERD and Khartoum for most months and seasons.

**Fig. 9:**
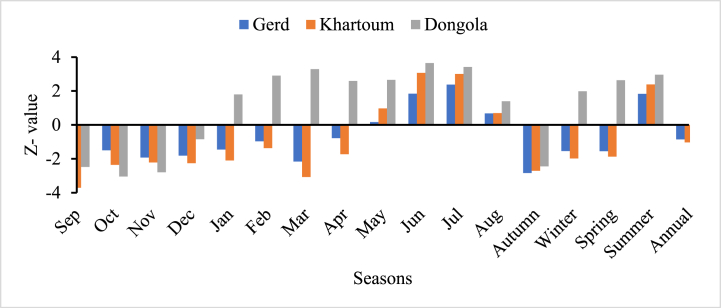
Monthly, seasonal, and annual hydrological drought trends in the Blue Nile basin for selected stations.

From Table 5 Mann – Kendell trend value, it is clear that the hydrological drought will be a threat in the future for all the riparian countries from September to December. For the last 102 years, the monthly trend indicates there will be a probability of recurrence of hydrological drought in the Autumn season for all of Ethiopia, Sudan, and Egypt (see Table 5 September–December value of z). therefore, climate change projection analysis is important to predict the future implications of the hydrological drought trend in the Blue Nile River basin (Fig. 9).

### 3.2 Discussion

Previous studies indicate that Ethiopia has been recurrently hit by severe and extreme meteorological droughts in the last five decades [50]. However, the studies are more focused on precipitation and temperature variability rather than their impact on agriculture and streamflow. The Blue Nile River basin has been frequently affected by moderate and severe drought for the last ten decades. However, the result of this study indicates the frequency of extreme drought was minimal for all selected stations. Dongola station is located downstream of Border (GERD) and Khartoum which is affected by five moderate, four severe, and two extreme drought events from 1900 to 2001. On the other hand, the most upstream station, Border is affected by 13 moderate, 3 severe, and 2 extreme drought events. Whereas, Khartoum station indicates the area was affected by 10 moderate, 6 severe, and 2 extreme drought events in the last 102 years. This result revealed that the upstream part of the basin (Ethiopia and Sudan) is more frequently affected by moderate drought than the downstream part of the basin (Egypt). This is because Egypt can receive more water both from the Blue Nile and White Nile while Ethiopia is the source of more tributaries and is directly affected by climate change.

The finding of this study is ideal with the previous studies in East Africa and the Blue Nile River basin [51,52]. Because the frequency and severity of hydrological drought in the Blue Nile Basin reduced as the time scale increased from monthly (SDI1) and seasonal (SDI3) to biannual (SDI6) and annual (SDI12). Even though the previous drought monitoring studies in the upper Blue Nile are mostly focused on meteorological drought analysis using SPI, RDI, SPEI, etc. their finding also approved that the severity and frequency of drought reduced as the time scale increased but the duration of drought to end is increased [53,54]. However, the recurrence interval is different from researcher to researcher findings, the common one is the trend of drought in Blue Nile has increased from decade to decade due to climate change in the region as well as in the globe [[55], [56], [57]]. In this study, it is understood that hydrological drought has an increasing trend in the Autumn (September–November) season in all states (Ethiopia, Sudan, and Egypt).

Climate change has a recurring impact on the hydrological system and it is a threat to many countries in the future [58,59]. SWAT and HBV models have been used to assess the impact of climate change on water resources and the consequence of hydrological drought on reservoirs using observed and projected climate data [60,61]. Recently, artificial intelligence and deep learning have become a good tool for hydrological drought forecasting using the Global Climate Model (GCM) and Regional Climate Models (RCM) [[62], [63], [64], [65]].

Most hydro-climate models are established to predict the future concentration of greenhouse gases (GHGs) based on the investigation of societal, political, and economic aspects that govern the emission scenario and land use land cover changes [[66], [67], [68], [69]]. But the real system in nature follows the actual course whereas the models simulate the possible courses of internal variability based on observed climate characteristics.

However, due to imperfect knowledge of future climate conditions and representation of the expected problems, two basic uncertainties developed in climate data projection. These are Knowledge uncertainty and Intrinsic uncertainties inherent to the problem [70]. From these two basic sources of uncertainties, three fundamental uncertainties are developed in climate change projection; scenario uncertainty (GHG emission scenario), model interaction uncertainty (model configuration and bias), and internal variable uncertainty (non-linearities of the climate system) [66,[70], [71], [72], [73], [74]]. Therefore, unlimited sources of uncertainties in climate change projection led to a cascade of uncertainty which also leads to an increasing envelope of uncertainties in future resource management strategies. For all those uncertainties, the time horizon of the projection, the scale of projection interest, and the variables under consideration are the major factors of uncertainty development in climate change projection. Besides these uncertainties, downscaling uncertainty is also important for variables mainly affected by a local process such as precipitation distribution and evapotranspiration process.

Another common uncertainty in developing countries is data scarcity which leads researchers to use secondary data and interpolation data. For example, the streamflow data for this study is interpolated from the nearby gauged station to the site of interest in the main river of the Blue Nile which causes quality uncertainties. So, accounting for the error of such uncertainties in quantity and quality in addition to climate data projection needs detailed research.

Therefore, within such uncertainties, global scientists are forced to use climate change projection models based on the following assumptions: (1) the full climate system is complex and it is difficult to represent the system analytically and statistically, (2) the future climate condition cannot be easily observed, and (3) the present anthropogenic climate forcing combined with the present-day climate baseline is a unique development of the earth's climate system [75]. In general, based on previous studies, model configuration, and GHG emission scenario uncertainties are more dominant for long-term climate change projection, especially on a global scale. Whereas, the contribution of climate system internal variability uncertainty is increased for short-term projections and advanced-order climate statistics [70,[76], [77], [78]].

Translating the meteorological variable such as precipitation to a hydrological variable (streamflow) using hydrological models constitutes a minor but not negligible uncertainty [66]. Therefore, hydrological extremes such as floods and droughts forecasting using downscaled climate data will have a sort of uncertainty. However, to develop a desired early warning system for drought mitigation measures in the future, climate change projection models simply play a great role. Recently, artificial intelligence (artificial neural network) and machine learning have become the most important programs to analyze historical trends of hydroclimatic conditions (dryness and wetness) and to forecast the future in different scenarios. Uncertainty developed from interpolated data leads to missing conclusions and decisions of researchers and policymakers on the development of water resource infrastructures. In addition to modern satellite information, it is also important to upgrade and install new ground-observed data instruments in such transboundary rivers collaboratively with riparian countries.

The rapid growth in agriculture, water supply, and other activities related to water consumption has created water scarcity issues worldwide. Simultaneously, climate change has projected more frequent and severe droughts are likely to occur [79,80]. Therefore, adaptive water resource management strategies are suggested for better-coordinated surface water and groundwater resources which are highly susceptible to hydrological drought. The riparian countries have to work hand in hand with adaptive water resource management strategies.

## 4 Conclusion

The phenomenon of drought constitutes a pressing natural hazard that triggers gradual yet far-reaching socio-economic repercussions within specific regional and national contexts. Despite the prominent consideration afforded to meteorological drought monitoring in both developed and developing nations, the analysis of hydrological drought remains relatively understudied, particularly in the East African region. In the Horn of Africa, the extensive rainfall variability coupled with the unreliable distribution of precipitation provokes considerable analytical challenges for characterizing meteorological drought within extensive areas, such as the Blue Nile River basin. Conversely, the use of long-term streamflow data for longer rivers offers valuable insights into the hydrological drought's effects on fundamental infrastructure such as hydropower, irrigation, and water supply projects, thereby providing critical information for multiple riparian nations. Consequently, a comprehensive hydrological drought monitoring and trend analysis was conducted within the Blue Nile River Basin. Throughout this endeavor, historical streamflow data obtained from the Ministry of Water and Energy, Ethiopia, for three stations spanning the period from 1900 to 2001, comprised the primary dataset. Using the streamflow drought index (SDI) on four distinct time scales; SDI1, SDI3, SDI6, and SDI12, this investigation aimed to clarify the characteristics of historical hydrological drought conditions. The results disclosed that the years 1912/13 and 1913/14 represented the most severe instances of drought for all selected stations, with an additional extreme drought occurrence noted in the year 1986/87 for Khartoum station. Upon analyzing the trend of drought occurrences over the past 102 years, it became evident that a total of nearly four severe and two extreme drought years transpired within the Blue Nile River basin, corresponding to probabilities of occurrence of 3.9 % and 1.9 %, respectively. Moreover, trend analysis revealed a statistically significant increasing trend in drought severity during the autumn season for all stations under consideration and a noticeable increasing trend of wet conditions in the remaining three seasons. On the other hand, the annual trend analysis did not show significant trends across all selected stations. Ultimately, this study underscores the imperative necessity of engaging in appropriate and integrated water resource management practices within upstream countries to mitigate the development of hydrological droughts, which may eventually give rise to socio-economic drought occasioned by supply-demand imbalances. Consequently, riparian nations must partake in Ethiopia's Green Legacy initiative to mitigate the worst-case hydrological drought scenarios while also contributing to the establishment of soil and water conservation structures within the Ethiopian highlands, the primary source of the Blue Nile River's flow. Furthering this line of inquiry, professionals in riparian countries are encouraged to prioritize pragmatic, technical issues pertinent to climate change and natural flow conservation, rather than engaging in discussions centered on hypothetical or impracticable concerns. In this context, expert consensus opines that effective adaptation and early warning systems must be established to mitigate the impacts associated with drought and floods during extreme hydrological events. This study's findings are intended to inform and guide decision-makers, water resource managers, and researchers engaged in research and policy formulation with adequate data on the hydrological drought severity and trend within the Blue Nile River basin. Lastly, the present research underscores the imperative necessity for future researchers to emphasize the holistic monitoring and predictive analysis of meteorological and hydrological drought within upstream countries (Ethiopia) as a means of enhancing region-wide resilience and reducing risk in the face of these pivotal threats. Hydrological drought monitoring is more important to downstream countries Egypt and Sudan because precipitation in those countries is erratic and it is difficult to analyze meteorological drought.

## Data availability statement

Data will be made available on request.

## Declaration of competing interest

I declared that there is no conflict of interest in this research paper.
